# “Who am I going to stay with? Who will accept me?”: family‐level domains influencing HIV care engagement among disengaged adolescents in Kenya

**DOI:** 10.1002/jia2.25890

**Published:** 2022-02-22

**Authors:** Courtney Myers, Edith Apondi, Judith J. Toromo, Mark Omollo, Salim Bakari, Josephine Aluoch, Festus Sang, Tabitha Njoroge, Zariel Morris, Rami Kantor, Paula Braitstein, Winstone M. Nyandiko, Kara Wools‐Kaloustian, Batya Elul, Rachel C. Vreeman, Leslie A. Enane

**Affiliations:** ^1^ Division of Infectious Diseases Department of Medicine Indiana University School of Medicine Indianapolis Indiana USA; ^2^ Academic Model Providing Access to Healthcare (AMPATH) Eldoret Kenya; ^3^ Moi Teaching and Referral Hospital Eldoret Kenya; ^4^ The Ryan White Center for Pediatric Infectious Disease and Global Health Department of Pediatrics, Indiana University School of Medicine Indianapolis Indiana USA; ^5^ Indiana University‐Purdue University‐Indianapolis Indiana University Indianapolis Indiana USA; ^6^ Division of Infectious Diseases Department of Medicine Brown University Apert Medical School Providence Rhode Island USA; ^7^ Department of Epidemiology Indiana University Fairbanks School of Public Health Indianapolis Indiana USA; ^8^ Division of Epidemiology Dalla Lana School of Public Health University of Toronto Toronto Ontario Canada; ^9^ Department of Medicine School of Medicine, College of Health Sciences Moi University Eldoret Kenya; ^10^ Department of Child Health and Pediatrics School of Medicine, College of Health Sciences Moi University Eldoret Kenya; ^11^ Indiana University Center for Global Health Indianapolis Indiana USA; ^12^ Department of Epidemiology Mailman School of Public Health Columbia University New York New York USA; ^13^ Department of Health System Design and Global Health Icahn School of Medicine at Mount Sinai New York New York USA; ^14^ Arnhold Institute for Global Health New York New York USA

**Keywords:** adherence, adolescents, HIV care continuum, retention, stigma, structural drivers

## Abstract

**Introduction:**

Adolescents living with HIV (ALHIV, ages 10–19) have developmentally specific needs in care, and have lower retention compared to other age groups. Family‐level contexts may be critical to adolescent HIV outcomes, but have often been overlooked. We investigated family‐level factors underlying disengagement and supporting re‐engagement among adolescents disengaged from HIV care.

**Methods:**

Semi‐structured interviews were performed with 42 disengaged ALHIV, 32 of their caregivers and 28 healthcare workers (HCW) in the Academic Model Providing Access to Healthcare (AMPATH) program in western Kenya, from 2018 to 2020. Disengaged ALHIV had ≥1 visit within the 18 months prior to data collection at one of two sites and nonattendance ≥60 days following their last scheduled appointment. HCW were recruited from 10 clinics. Transcripts were analysed through thematic analysis. A conceptual model for family‐level domains influencing adolescent HIV care engagement was developed from these themes.

**Results:**

Family‐level factors emerged as central to disengagement. ALHIV‐particularly those orphaned by the loss of one or both parents‐experienced challenges when new caregivers or unstable living situations limited support for HIV care. These challenges were compounded by anticipated stigma; resultant non‐disclosure of HIV status to household members; enacted stigma in the household, with overwhelming effects on adolescents; or experiences of multiple forms of trauma, which undermined HIV care engagement. Some caregivers lacked finances or social support to facilitate care. Others did not feel equipped to support adolescent engagement or adherence. Regarding facilitators to re‐engagement, participants described roles for household disclosure; and solidarity from caregivers, especially those also living with HIV. Family‐level domains influencing HIV care engagement were conceptualized as follows: (1) adolescent living situation and contexts; (2) household material resources or poverty; (3) caregiver capacities and skills to support adolescent HIV care; and (4) HIV stigma or solidarity at the household level.

**Conclusions:**

Family‐level factors are integral to retention in care for ALHIV. The conceptual model developed in this study for family‐level influences on care engagement may inform holistic approaches to promote healthy outcomes for ALHIV. Developmentally appropriate interventions targeting household relationships, disclosure, HIV stigma reduction, HIV care skills and resources, and economic empowerment may promote adolescent engagement in HIV care.

## 1 INTRODUCTION

There are an estimated 1.7 million adolescents living with HIV (ALHIV, ages 10–19) globally [1]. Despite global scale‐up of antiretroviral therapy (ART), ALHIV experience poorer outcomes in the HIV care cascade as compared to other age groups, including lower viral suppression and retention in care [2, 3, 4, 5]. Consequently, ALHIV have not had the same declines in mortality as other age groups [6]. Dedicated efforts are needed to improve outcomes for this particularly vulnerable group [7, 8, 9, 10].

ALHIV are a unique population. Developmentally, they are gaining independence and navigating social relationships, but they also depend on caregivers to support their HIV care [8, 11, 12, 13]. Previous studies have examined caregiver challenges in HIV care, often without differentiating younger children from adolescents. Barriers experienced by caregivers include stigma, isolation, poverty and depression, and challenges caring for orphaned children or adolescents [14, 15]. By contrast, family‐level contexts specifically for adolescent HIV care are often overlooked. ALHIV may be treated in paediatric or adult services, and may have limited support during developmental and care transitions, including to achieve self‐management skills [16, 17, 18]. ALHIV experience developmentally specific influences and transitions related to caregiver, peer and social relationships, and economic needs or support, which impact their retention, adherence and wellbeing [12, 13, 19, 20, 21, 22]. There is a need to integrate an understanding of family‐level environments, relationships and contexts for adolescent engagement in HIV care [21, 23]. Otherwise, currently conceptualized adolescent‐friendly services may be insufficient to meet the needs of ALHIV who may be highly vulnerable due to adversities experienced at the family level [13, 24].

In this qualitative study, we assessed family‐level factors central to disengagement or supporting re‐engagement of adolescents in HIV care, through in‐depth interviews with disengaged ALHIV, their caregivers and healthcare workers (HCW). Findings informed the development of a conceptual model for family‐level influences on adolescent engagement in HIV care. This was interrogated alongside a “theories of practice” framework, with elaboration of adolescent developmental contexts for HIV care engagement [25].

## 2 METHODS

### 2.1 Study design

We conducted this qualitative analysis of family‐level factors influencing adolescent disengagement as part of a broader study of disengaged ALHIV in the Academic Model Providing Access to Healthcare (AMPATH), which has been described previously [12].

### 2.2 Study setting

AMPATH is a partnership between Moi University, Indiana University, a consortium of academic institutions and the Ministry of Health in Kenya [26, 27, 28, 29]. ALHIV have access to adolescent‐friendly services at the dedicated Rafiki Center for Excellence in Adolescent Health at Moi Teaching and Referral Hospital (MTRH) in Eldoret. A range of adolescent‐friendly services are available at other AMPATH sites throughout western Kenya.

On enrolment in care and subsequent visits at AMPATH sites, patients are informed of and consent to standard outreach and tracing practices; detailed contact and locator information are maintained [30, 31, 32]. The outreach team partners with community health workers in robust efforts to follow‐up, trace and re‐engage patients through phone calls and/or home visits within days after a missed appointment or after a gap in care [30, 31, 32].

### 2.3 Participant recruitment

ALHIV who had disengaged from HIV care were included using the following criteria: ALHIV ages 10–19 at their last clinic visit; enrolled in HIV care at MTRH or Kitale; attended clinic ≥1 visit within the 18 months prior to data collection; and subsequently absent from clinic for ≥60 days following a missed appointment [12]. The research team worked closely with the outreach staff and community health workers to trace disengaged ALHIV and facilitate re‐engagement, through standard approaches described above [12]. Adolescents were excluded if tracing revealed that they had transferred care without any period of disengagement. Eligible ALHIV and caregivers were offered participation in this study. Interviews with ALHIV and their caregivers were conducted from May 2019 to March 2020. A purposive sample of 42 disengaged ALHIV and 34 of their caregivers achieved saturation of themes for the broad study of multilevel barriers and facilitators to care and reasons for disengagement.

HCW working with ALHIV were purposively sampled for qualitative interviews, to include a range of roles in 10 clinic sites with varied settings and availability of adolescent‐friendly services, from October 2018 to January 2019. A purposive sample of 28 HCW‐11 clinical officers, five nurses, eight outreach workers, three social workers and one psychologist‐reached saturation of themes surrounding the above research questions.

### 2.4 Ethics

Appropriate human subject protections were used throughout the study procedures. Adult participants provided informed consent and adolescent minors provided assent, with consent of their primary caregiver. Participants were informed about the potential sensitivity of questions; that any safety concerns identified would require disclosure to appropriate clinic staff; and that they could decline to answer any questions or to participate at any point. ALHIV were encouraged and facilitated by the study team to re‐engage in HIV care and were referred to care resources as needed. The research staff facilitated in‐the‐moment support for issues identified in interviews, including through peer mentor or social work support, and referral to other existing resources within the clinic. Research staff directly liaised with appropriate clinical staff when safety concerns were identified, for further evaluation, management and/or reporting under established clinic procedures. The research team followed up with adolescents/caregivers after interviews to ensure that unmet needs were addressed.

The study protocol was reviewed and approved by the Institutional Research and Ethics Committee, constituted jointly by Moi University College of Health Sciences and MTRH, and by the Institutional Review Board at Indiana University.

### 2.5 Interviews

Semi‐structured qualitative interview guides were developed based on the research questions and socio‐ecological framework guiding the broader study, which incorporated adolescent developmental and care transitions [2, 8, 13]. Questions were open‐ended and assessed multilevel barriers and facilitators to retention, including a set of dedicated questions regarding family‐level factors related to disengagement or supporting potential re‐engagement in care. A separate interview guide that did not mention HIV was used with adolescents who on screening did not disclose awareness of their HIV status [13].

While participants were given the option to conduct study consent or interviews at home or at a private location of their choice, all chose to complete interviews in a private space in their clinic or in the research office. Interviews were performed by research staff who are fluent in Swahili and English, with extensive training and experience working with ALHIV and performing qualitative interviews. The research staff had no prior engagement with adolescent/caregiver participants. Interviews were conducted separately for adolescents and caregivers. Interviews were audio‐recorded and translated/transcribed into English, and translations were independently verified.

### 2.6 Analysis

A codebook was developed and thematically organized around the research questions and socio‐ecological framework [13]. Initial transcript analysis facilitated elaboration of the codebook, which was then iteratively refined through discussion of the research team. Multiple team members independently coded initial transcripts to establish inter‐rater reliability. Transcripts were then coded by multiple team members working independently and using the Dedoose platform (SocioCultural Research Consultants, Los Angeles). Code reports were developed for interrogation of data regarding family‐level factors underlying disengagement and resources for re‐engagement in care.

### 2.7 Conceptual model

Through close reading of qualitative data using constant comparison across participant groups and perspectives, overarching themes emerged. These were organized into central domains underlying retention or disengagement from care at the level of the household or other environments in which adolescents live.

Findings were then interrogated alongside a “theories of practice” framework for engagement with HIV services [25]. This framework posits that elements of HIV care engagement may be organized into life practices, conceptualized as “materiality,” “competence,” “meaning,” and “other life practices” [25]. These elements influence one another and may differ over time and across settings [25, 33]. We examined our findings through this lens, and reflected on any needs for expansion of this framework for adolescent developmental transitions and family‐level contexts for HIV care engagement.

## 3 RESULTS

**Figure 1:**
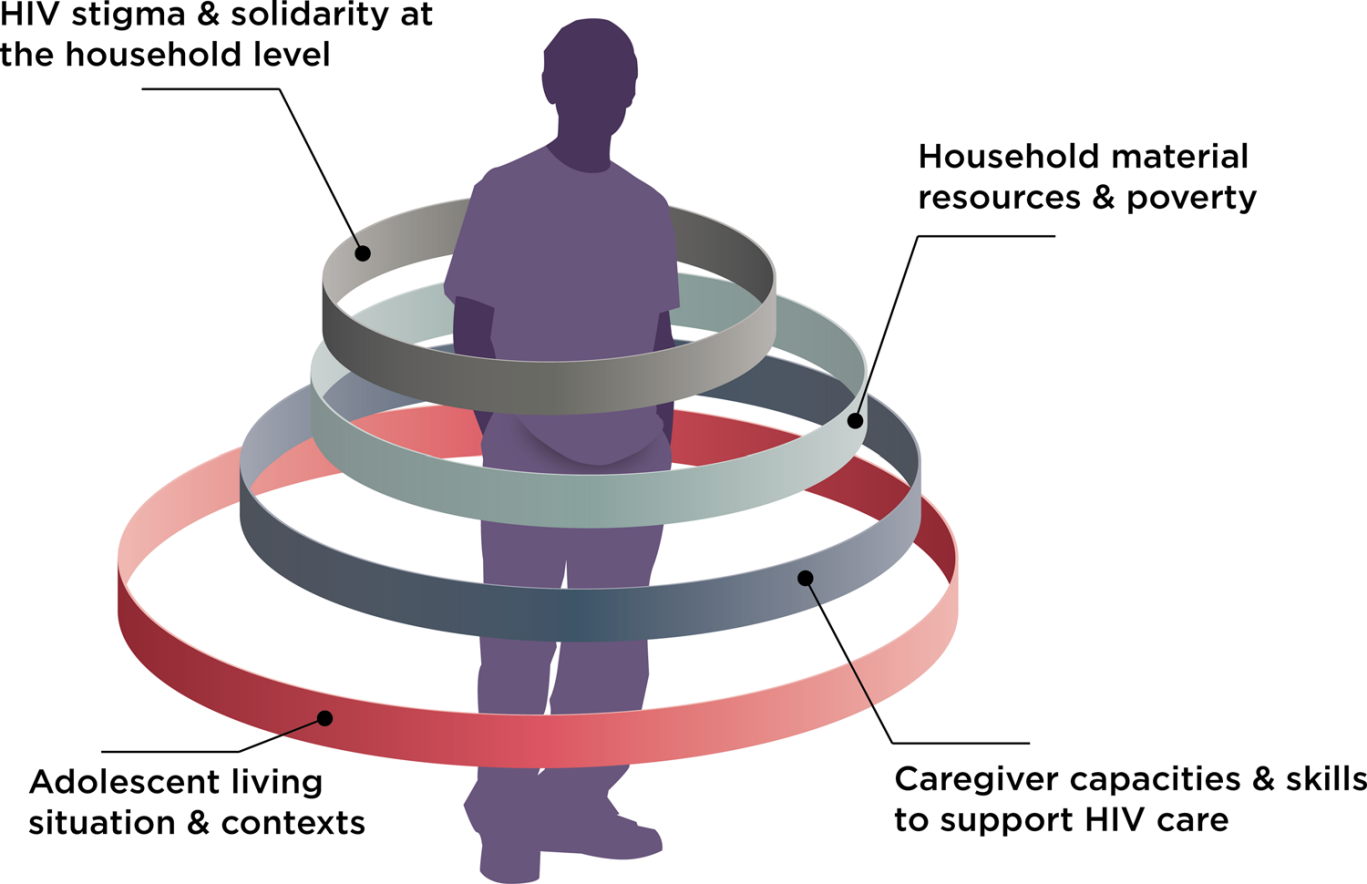
Conceptual model for family‐level domains influencing adolescent HIV care engagement.

Demographic characteristics of disengaged ALHIV are presented (Table 1). Themes are described for family‐level factors related to disengagement, or resources for re‐engagement, in HIV care. Through detailed analysis of participant narratives, central domains emerged, which were organized within a conceptual model for family‐level influences on adolescent HIV care engagement (Figure 1). These domains centred on: (1) adolescent living situation and contexts; (2) household material resources or poverty; (3) caregiver capacities and skills to support adolescent HIV care; and (4) HIV stigma or solidarity at the household level.

**Table 1:** Demographic characteristics of adolescents disengaged from HIV care included in this study

Variable	Adolescents
	*N* = 42 (%)
Age (years) at last kept visit	
10–14	15 (35.7)
15–19	27 (64.3)
Sex	
Female	26 (61.9)
Male	16 (38.1)
Orphan status	
Both biological parents living	14 (33.3)
Mother deceased only	13 (31.0)
Father deceased only	6 (14.3)
Both biological parents deceased	9 (21.4)
Adolescent's primary caregiver	
Mother	18 (42.8)
Father	5 (11.9)
Uncle or aunt	9 (21.4)
Grandmother	4 (9.5)
Sibling	2 (4.8)
Other	4 (9.5)
Current living situation	
Household with immediate family (includes extended family)	26 (61.9)
Household with extended family (with no immediate family)	11 (26.2)
Household with non‐family members	1 (2.4)
Institution	3 (7.1)
Homeless or living on the street	1 (2.4)

### 3.1 Adolescent living situations and contexts

Heterogeneous living situations and contexts emerged as important settings for adolescent HIV care. Underlying instability in adolescent living situations was a central factor in disengagement. This included changes in residence or caregivers, or adolescents or caregivers leaving home. Orphaned adolescents faced particular challenges navigating HIV care while also coping with parental loss, relying on new caregivers, changing households or living on the street.

“*It's like she is asking herself, ‘where am I going and I don't have a mother? Who am I going to stay with? Who will accept me?*” – Caregiver of 20 year‐old female

Adolescents were vulnerable to disengagement when new caregivers lacked information or resources to support their care. Some new caregivers or household members had not been disclosed to regarding the adolescent's status, given anticipated stigma.

“*I went to these family members that I have never gone to [before]… It's far from town, something like a rural area, then I found it hard because they never knew my status*.” – 20 year‐old female

ALHIV who had run away, moved transiently or were street‐connected became disengaged due to lacking support or stability for HIV care, and anticipated stigma from peers.

“*Because when [I] was with my friends [on the street], I did not want them to know that I am taking drugs [ART]. So, I stayed for three to four months without taking the drugs. I felt weak and I was being looked for by my mother. She was being told that I am sick. When I would go back home, I would take the drugs for about two months and then go back to my friends*.” – 19 year‐old female

Further challenges included when adolescents relocated to rural areas, moved to boarding school or when caregivers needed to leave home for work.

“*I was just staying here, so I was just coming on foot. It was near, but now that dad retired last year, he went to [rural town]. So, from there is when transport has been an issue*.” – 19 year‐old male

While unstable living situations contributed to disengagement, having supportive caregivers and clinic‐level flexibility supported re‐engagement in care, such as by accommodating scheduling needs or transfer to an accessible site.

“*We advised her to come [home to a different caregiver] and take a transfer so that she could come [to clinic] nearby, because we thought coming far could have been discouraging her. So, if she came nearby, it would be easy for her because it is a place where, even if she does not have bus fare, she can walk and get there. So, since she came back and as we have been close to her to monitor her, I see there are changes*.” – Caregiver of 20 year‐old female

### 3.2 Household material resources and poverty

Household poverty was a frequent barrier to retention, when families lacked funds for transportation to clinic, food to take with ART or basic needs. As one HCW described, some patients “*have become lost to follow‐up just because of lack of food.”*

“*I do home visits… You can go to some places and you even feel traumatized. You get to someone's place and there is nothing, not even a seat. I mean you feel for sure that this one needs to be helped*.” – HCW

Elderly caregivers particularly struggled to support transportation costs. Some adolescents disengaged after an emergency, such as a family member's illness, that exhausted the family's means.

“*It was fare only. My husband was sick. My son got an accident, and his leg was amputated. So, we lacked any means, and it was a problem. That was the time I lacked money. Any little that we could get, we could bring it to the hospital. She took two months before she could come. She came on the third month. Maybe they even said that she has stopped being in clinic*.” – Caregiver of 17 year‐old female

Conversely, financial resources facilitated adolescent re‐engagement. Caregivers who were able to save funds and arrange absences from work could support adolescents’ appointments.

“*We have been given the appointments so you just know that a certain date we have to dedicate that day, even if there is some work to be done, I'd rather close the work, like today I have not opened my work*.” – Caregiver of 15 year‐old female

One orphaned adolescent who had disengaged after lacking support from caregivers worked to earn income to return to care.

”*I went and upon reaching there, I stayed for days and then I started thinking about [ART]. I told my sister, ‘let us do some casual jobs and get some money so that I can go to the hospital’. We did the work and she gave me the money and then I came*.” – 17 year‐old female

Other adolescents sought support from family members for transportation costs. Having disclosed their HIV status to relatives facilitated this support.

“*At times when I lack fare, I would go and ask them, ‘help me with fare to town’ and everyone would ask, ‘what are you going for?’ I would just tell them, ‘I am going for treatment in the clinic,’ and they would contribute like fifty shillings each and I get the amount I need, then come*.” – 20 year‐old female

### 3.3 Caregiver capacities and skills to support HIV care

Some participants noted that ALHIV disengaged after caregivers were unable to supervise or help navigate care.

“*He was not keen with taking her to the clinic or to encourage her to come to the clinic. He used to work as a mobile electrician, so sometimes he would be in Nairobi, he was mostly on call. The wife mostly was not following up on the child's clinic*.” – Caregiver of 17 year‐old female

Some orphaned adolescents disengaged after moving to caregivers with limited knowledge about HIV or its management.

“*Some of them; and especially those who are challenged in a way, maybe those who are orphaned and they are being taken care of by guardians, uncles… maybe the parents died and now they are being taken care of by an uncle who may not really understand the nature of this disease*.” – HCW

Disengagement also resulted when caregivers had limited capacity to support care. Such situations were compounded by household poverty, for example when caregivers needed to leave home for work.

“*I was not there. I had gone to Uganda to my sister… Whenever I could ask him about it, he always told me, ‘Mum, money is the problem, I don't know what and what,’ but I would tell him to work hard and look for a means to go back to the clinic… So that is what brought him down. I was not around to follow him up if he was taking his drugs or not*.” – Caregiver of 17 year‐old female

Additional situations similarly resulted in adolescents having autonomy in care before they were ready, contributing to disengagement.

“*It got to a place I thought, she's a grown up, she can do her things for herself. That's where she went and got lost*.” – Caregiver of 20 year‐old female

Many caregivers described lacking social and mental health support to care for ALHIV. One caregiver described frustrating and isolating experiences caring for an adolescent who had run away, and how it “became too much for [her].”

“*There is a time we were to come with him, but he ran away. That thing [bothered] me so much I lost my breath, I could not speak, and I said I would never come back here again. I even told them I would not come back here again; I think I will die here for no reason. I think I was very angry, because I could not breathe nor speak. I wondered what was wrong with me… He went back to the streets and refused [to participate in care]… I just let it be for the time being. It bothers me inside, but I can't tell anyone*.” – Caregiver of 19 year‐old male

By contrast, caregiver capacities to help navigate HIV care facilitated engagement. This included supervising ART adherence, reminding the adolescent of appointments, accompanying them to clinic or being in communication with the clinic.

“*He says he came to the clinic, I make sure … I have seen the card and I have seen the medication. So that way I know he has been to the clinic*.” – Caregiver of 16 year‐old male

Disclosure of the adolescent's status to relatives further enabled such support.

“*[The family's awareness of my HIV status] has made it easy. Because since they know it's even them who tell me, ‘don't forget to go the clinic, you should always go’*.” – 18 year‐old female

### 3.4 HIV stigma and solidarity at the household level

Enacted or anticipated stigma in the household was a major barrier among disengaged adolescents, particularly among orphaned ALHIV. Because having family support was critical for ALHIV navigating widespread HIV stigma in the community, experiencing stigma and isolation within the household was particularly damaging and contributed to disengagement from care.

#### 3.4.1 Anticipated stigma and nondisclosure within the household

For adolescents settling into new households, anxiety surrounding HIV status disclosure and anticipated stigma prevented them from seeking resources to continue in care.

“*Telling them I want to go to the clinic, they will ask like, ‘what is the problem?’ And I like keeping my things confidential. Then I was like, I cannot tell them, because now I will start explaining everything from the start. So, I just kept quiet until my clinic day passed*.” – 20 year‐old female

Incomplete household disclosure contributed to disengagement, including when the adolescents themselves were unaware of their HIV status.

“*There is a time the parents disagreed, and the mother had to leave that home and this particular child remained but with a busy father and no one else wanted to take the child to the clinic because honestly they didn't know. The siblings are asking this child, ‘Nowadays you don't take the medication you used take? What happened?’ The small child had not been disclosed his status*.” – HCW

Issues surrounding disclosure to adolescents, including inadvertent or delayed disclosures, or unresolved concerns for adolescents coping with disclosure, contributed to adolescent and caregiver conflicts, mental health burdens and disengagement from care.

“*I feel so bad to the extent that I don't want the drugs. I even blame them [my parents] for infecting me*.” – 20 year‐old female

When adolescents were the only ones in the family that they knew to be living with HIV, they felt further isolated.

“*I have other cousins living with grandma, of the same age, we are in the same class. So, you find it a challenge finding that you are the only one taking medication, you ask yourself why it's just me*.” – 16 year‐old male

#### 3.4.2 Enacted stigma at the household level

Enacted stigma from caregivers greatly impacted adolescent wellbeing and HIV care engagement. Narratives of caregiver stigma detailed social isolation, rejection and abuse.

“*When he was a little boy, after the death of the mother, when he was taken by the stepfather to Nairobi… they were never given food, they were discriminated against, they were sick, they were never taken to the hospital, until the neighbours raised alarm*.” – Caregiver of 14 year‐old male

#### 3.4.3 Solidarity and emotional support from caregivers and family

Caregivers who demonstrated solidarity with adolescents were able to support re‐engagement in HIV care and engender a sense of security and hope.

“*The thing I can say makes it [easier] for her to come to clinic is me and the mother. Because*

*we understand her, we understand her situation and we do things as a team*.” – Caregiver of 15 year‐old female

Household and clinic support were critical for adolescents in accepting their diagnosis and re‐engaging in care.

“*My first reaction was, I was thinking of ending my life, but through guiding and counseling [from family and clinic], I agreed to accept my status and to start medication*.” – 17 year‐old male

Adolescents were strongly influenced by having role models in their families who were thriving with HIV.

“*I also see my mother taking them. So, when I see her taking the drugs, I also get that courage to take those drugs… I will have seen that my mother went [went to clinic] and I should also go*.” – 20 year‐old female

Caregiver and household emotional and social support engendered hope among adolescents, and in turn, supported care engagement.

“*These children should not feel, ‘why don't I have someone to talk to me?’ They should not be left alone. When we have come here and you have talked to her that gives this child some hope they feel like, ‘this one wants me to live’. These children should also be taken care of like other children*.” – 17 year‐old female

Supportive caregivers assured adolescents of confidentiality and solidarity as they helped them navigate care challenges and HIV stigma.

“*He just knows that we are together, and we deal with our issues together. So, when we talk about issues, we just talk the two of us*.” – Caregiver of 16 year‐old male

“*When people were talking about me, my mother told me to just go and not fear to take the drugs… My mother told me, ‘let them just talk.’*” – 16 year‐old female

## 4 DISCUSSION

This qualitative study lends key insights for understanding adolescent engagement in or disengagement from HIV care. The inclusion of disengaged ALHIV provided critical insights to understand drivers of and vulnerabilities to disengagement, and to map the sources of support that could be leveraged to promote re‐engagement. Further, this analysis was strengthened by triangulating the perspectives of adolescents, their caregivers and HCW. The comprehensive approach taken in this study facilitated a holistic understanding of family‐level contexts for HIV care among ALHIV. We noted that factors contributing to disengagement include changes in caregivers or living situations, household poverty, enacted stigma and barriers for caregivers to support adolescent HIV care and transitions to self‐management. Conversely, the presence of household resources, caregiver support in HIV management, family‐level solidarity and supported caregiver transitions may support adolescent re‐engagement. These emergent themes were organized within a conceptual model for family‐level domains influencing adolescent HIV care engagement.

Findings in this study point to a need to more fully integrate adolescent developmental transitions in adolescent HIV care and research, and to address family‐level and caregiver contexts in supporting adolescent transitions in care. Indeed, engagement challenges in this study appeared to have their greatest impact in late adolescence, as adolescents may need greater support in transitions towards self‐management [18, 20, 34]. We interrogated our findings alongside the “theories of practice” framework, and further expanded on it in considering adolescent developmental and caregiver contexts that emerged in this study (Table 2) [25, 33]. Adolescent engagement in HIV care has fundamental differences as compared to adults (previously addressed with this framework), including: heightened influence of caregiver or household resources and social determinants of health; developing adolescent autonomy; potentially greater influences of stigma; and reduced control over living situation or contexts. As such, we expand on the theories of practice framework to consider the family‐level domains that influence adolescents’ retention in HIV care, where interventions may bolster family‐level support for adolescent HIV care engagement. This understanding further aligns with recent research demonstrating the influence of family, peer and social support on adolescent transitions to autonomy and self‐management in HIV care [18, 20, 34]. Addressing family‐level contexts and support may, therefore, contribute to healthy adolescent transitions to self‐management and improved HIV care outcomes.

**Table 2:** Domains influencing adolescent HIV care engagement within the household/family level, with example factors within each domain

Organizing domains	Adolescent living situations and contexts	Household material resources and poverty	Caregiver resources and skills to support HIV care	HIV stigma and solidarity at the household level
“Theories of practice” [25]	“Other life practices”	“Materiality”	“Competence”	“Meaning”
Factors underlying disengagement				
	Orphan status	Household poverty	Caregivers unable to accompany adolescent to clinic	Anticipated stigma within household
	Change in caregivers	Food insecurity	Caregivers unable to supervise adherence/appointments	Non‐disclosure to household/family
	Leaving home/running away	Transportatio costs	Caregivers with limited HIV knowledge	Delayed/unsupported disclosures to adolescents
	Living on the street	Caregivers with limited finances, including elderly relatives	Adolescents expected to self‐manage HIV without support	Enacted stigma within household, abuse
	Mobility/shifting residences	Lacking emergency funds	Caregiver mental health burdens	Caregivers disclosing adolescent's status to others without consent or basis
	Residing at boarding school	Limited financial support when relatives not informed of adolescent's health needs		Coping with community/societal stigma with limited caregiver support
	Caregivers away from home	Caregivers away for work	Caregivers away for work	
Factors supporting re‐engagement				
	Supportive caregivers	Financial reserves	Accompaniment to clinic	Emotional support/solidarity
	Caregivers engaged in HIV care	Ability to take time from work to support care	Supervision of adherence, appointments	Family members living with HIV
	Adaptability of clinic/HCW to caregiver transitions/challenges	Financial support from disclosed relatives	Caregiver support for transitions to adolescent self‐management	Reassurance and orientation towards future thinking
			Disclosure to supportive relatives	Caregiver confidentiality and trust
				Support for overcoming stigma in the community/society

Note: Examples are provided within each domain, and in relation to the theories of practice framework [25].

This conceptual model illustrates key domains where family‐level interventions may support adolescent HIV care engagement. We consider existing evidence within these conceptual domains.

Across adolescent living situations and contexts, this study found that orphaned ALHIV have distinct vulnerabilities to disengagement, which often related to transitions to new caregivers or transient living situations. Relatively few studies have specifically focused on the needs of orphaned ALHIV, who may have limited social and family support networks to support their retention and engagement in HIV care, and who may experience acute challenges in their environments [12, 13, 32, 35]. Orphaned ALHIV in Kenya experience marked mental health burdens, particularly when street‐connected or living with extended relatives [36, 37, 38]. Interventions targeting the needs of orphaned ALHIV may be needed.

Household solidarity is clearly impactful and should be a target for intervention. Family cohesion has been associated with improved treatment self‐efficacy in adults and ALHIV [21, 39]. Support from caregivers and siblings has been associated with reduced mental health challenges in ALHIV [19, 40]. A multilevel intervention of Together for Empowerment Activities based in social interdependence for adults with HIV and family members resulted in reduced depressive symptoms and improved coping skills [41]. Interventions in HIV‐affected households have resulted in reduced depression in children, and reductions in intimate partner violence and harmful alcohol use in caregivers [42, 43]. Family solidarity or stigma‐reduction interventions to promote adolescent HIV care engagement should be studied. This might include family therapy or interventions targeting household stigma. Particular challenges at the adolescent stage include needs for supported disclosure to adolescents, and to provide ongoing supervision of care while supporting transition to independence. Caregiver interventions are needed to optimally support such developmental transitions.

Poverty is a major barrier to adolescent HIV care engagement and wellbeing [19]. Studies have examined financial interventions‐including cash transfers, incentives and savings programs‐targeting child and adolescent health and HIV outcomes [35, 44, 45, 46, 47, 48, 49, 50]. The Suubi+Adherence family economic empowerment intervention consisting of child development savings accounts combined with microenterprise workshops was associated with viral suppression among ALHIV with previously detectable viral load at baseline [35]. Our findings emphasize the potential for such approaches to economic empowerment, particularly if combined with care resources, skills or trainings further supporting care engagement, to promote adolescent health and optimal HIV outcomes for the most vulnerable ALHIV.

This qualitative study allowed for in‐depth examination of family‐level factors influencing adolescent engagement in HIV care from the perspectives of disengaged adolescents, caregivers and HCW. Findings are further validated by their resonance with an established health behaviour framework, and alignment with adolescent barriers and facilitators to retention observed in other studies [2, 8, 11, 12, 13, 21, 25, 32, 51, 52, 53]. A limitation of this study is that engaged ALHIV were not included in this analysis; however, our findings regarding facilitators to retention align with these prior investigations, which included engaged ALHIV. We also note that few street‐connected adolescents were included in this sample. Street‐connected adolescents may have additional challenges and barriers to retention in care beyond those identified here for orphaned adolescents living in other contexts [31, 32].

## 5 CONCLUSIONS

Family‐level factors are integral to retention in care for ALHIV. Working with disengaged ALHIV, caregivers and HCW, we developed a conceptual model for family‐level influences on engagement in HIV care. These areas included adolescent living situations and contexts, material resources, caregiver skills and capacities to support care, and perhaps most crucially, the presence of either stigma or solidarity at the family level. Findings are further validated by their alignment with an established theoretical framework, and inform holistic approaches to intervention at the family or household level for ALHIV. Developmentally appropriate interventions targeting household relationships, disclosure, HIV stigma reduction, care resources and economic empowerment may promote adolescent engagement in HIV care.

## COMPETING INTERESTS

The authors declare no competing interests.

## AUTHORS’ CONTRIBUTIONS

LAE, EA, WMN, PB, KWK and RCV conceptualized this study. EA, JA, FS, TN, RK, KWK, BE, RCV and LAE partner in a parent study which identified, traced and supported the re‐engagement of disengaged adolescents. MO and SB recruited and enrolled participants and performed interviews. LAE, EA and JA supervised the study. LAE, EA, MO, JJT, SB, ZM and CM coded and analysed transcripts. CM and LAE performed all stages of the analysis and drafting of the manuscript. All authors participated in the analysis and revision of manuscript drafts. All authors have reviewed and approved the final manuscript.

## FUNDING

Research reported in this publication was supported by the Eunice Kennedy Shriver National Institute of Child Health & Human Development of the National Institutes of Health under Award Number K23HD095778. Dr. Enane's, Dr. Wools‐Kaloustian's and Dr. Vreeman's time was supported in part by the East Africa International Epidemiology Databases to Evaluate AIDS (IeDEA) regional consortium, which is funded by multiple institutes of the National Institutes of Health, through award U01AI069911. Dr. Kantor was supported in part by K24AI134359 and P30AI042853. This work was additionally supported by the Indiana University‐Purdue University of Indianapolis (IUPUI) Life‐Health Sciences Internship Program, funded by the IUPUI Commitment to Excellence Fund.

## DISCLAIMER

The content of this manuscript is solely the responsibility of the authors and does not necessarily represent the official views of the National Institutes of Health.

## Data Availability

The authors are unable to share full interview transcripts given needs to protect the privacy of participants, and the stipulations by which participants gave informed consent for this research.
